# Determining the sample size for a cluster-randomised trial: Bayesian hierarchical modelling of the ICC estimate

**DOI:** 10.1186/1745-6215-16-S2-P229

**Published:** 2015-11-16

**Authors:** Svetlana Tishkovskaya, Chris Sutton, Lois Thomas, Michael Leathley, Caroline Watkins

**Affiliations:** 1 University of Central Lancashire, Preston

## 

In common with many cluster-randomised trials, it was difficult to determine the appropriate sample size for the planned trial of the effectiveness of a systematic voiding programme for post-stroke incontinence due to the lack of a robust estimate of the intra-cluster correlation coefficient (ICC). One approach to overcome this problem is a method of combining ICC values in the Bayesian framework (Turner et al. 2005). We adopted this approach and used Bayesian hierarchical modelling to estimate the ICC.

To obtain the ICCs to combine in the model, we performed a literature search and identified a number of relevant studies with ICC estimates. To allow for uncertainty in ICC estimates, the Bayesian hierarchical model includes separate weights for each study and each outcome: study weights represent the degree of relevance to study population and intervention, and outcome weights are similarly related to the planned trial’s primary outcome (frequency of urinary incontinence). Assigning weights is rather subjective and is a source of uncertainty in the model. To minimise this subjectivity, we performed an exercise by which a team of expert reviewers with a range of relevant expertise assigned weights for each trial and each outcome. These weights were incorporated into the model to combine the individual ICC estimates and construct a distribution of a targeted ICC estimate to inform the sample size.

This approach should provide a more robust estimate of an ICC for determining the sample size for a cluster-randomised trial as it combines relevant ICCs in Bayesian framework.

